# Phenylethanoid Glycosides: Research Advances in Their Phytochemistry, Pharmacological Activity and Pharmacokinetics

**DOI:** 10.3390/molecules21080991

**Published:** 2016-07-29

**Authors:** Zhenzhen Xue, Bin Yang

**Affiliations:** Institute of Chinese Materia Medica, China Academy of Chinese Medical Sciences, Beijing 100700, China; xuezhenzhen99@126.com

**Keywords:** phenylethanoid glycosides, novel structures, bioactivity, pharmacokinetics

## Abstract

Phenylethanoid glycosides (PhGs) are widely distributed in traditional Chinese medicines as well as in other medicinal plants, and they were characterized by a phenethyl alcohol (C_6_-C_2_) moiety attached to a β-glucopyranose/β-allopyranose via a glycosidic bond. The outstanding activity of PhGs in diverse diseases proves their importance in medicinal chemistry research. This review summarizes new findings on PhGs over the past 10 years, concerning the new structures, their bioactivities, including neuroprotective, anti-inflammatory, antioxidant, antibacterial and antivirus, cytotoxic, immunomodulatory, and enzyme inhibitory effects, and pharmacokinetic properties.

## 1. Introduction

Phenylethanoid glycosides (PhGs) are a class of water-soluble compounds widely distributed in traditional Chinese medicines (TCMs), as well as other medicinal plants. They have been detected in roots, stems, leaves, flowers, fruits and seeds without organ selectivity, while their concentrations in each organ may vary a lot [1,2]. As their names suggest, PhGs are characterized by a phenethyl alcohol (C_6_-C_2_) moiety attached to a β-glucopyranose/β-allopyranose via a glycosidic bond. The core structures are often abundantly decorated with substituents such as aromatic acids (e.g., caffeic acid, coumaric acid, cinnamic acid, ferulic acid, and isoferulic acid) and various saccharides (e.g., rhamnose, xylose, apiose, glucose, lyxose, allose and arabinose) through ester or glycosidic linkages, respectively. The outstanding activity of PhGs in diverse diseases proves their importance in medicinal chemistry research. Several reviews on PhGs regarding their isolation and purification, structure elucidation, chemotaxonomy and biotransformations, and pharmacological activities have been reported [3,4]. Recently, interest in PhGs has been growing, with a significantly increasing volume of literature describing PhGs′ novel structures, diverse bioactivities, and evident roles in the prevention and treatment of various human diseases as well as their pharmacokinetics having been reported. Such rich information prompted us to review papers on novel PhG structures, their pharmacological activities and pharmacokinetics published in the last decade.

## 2. Phytochemistry

Since a 2008 review [4], more than 100 new PhGs have been isolated and identified. Compared with the known PhGs reported in [4], some of the new ones differed in their core structures, while others differed in the number and/or position of the substituents. The new PhGs with a typical phenethyl alcohol (C_6_-C_2_) moiety attached to a β-glucopyranose/β-allopyranose are listed in Table 1.

In the table references for the first reports on specific PhGs as new compounds are given, and the plant sources and biological activities reported for the specific PhGs are also included. Most of the isolated new PhGs were glucopyranosides, and the allopyranosides, which are rarely found in the plant kingdom, were mainly isolated from *Magnolia officinalis* [32]. Generally, glucose, galactose, xylose, apiose, arabinose and rhamnose were the most frequently occurring saccharides, while lyxose only appeared in compound **82** from *Teucrium chamaedris* [45]. Aside from the most frequently occurring substituents at C-3/4/7 of the phenylethyl moiety, which were hydroxy and methoxy groups, a glucose moiety occurred at the C-4 of aglycone of compound **62** from *M. officinalis* [32]. Additionally, the most frequently occurring aromatic acids that form esters with the glucose/allose were caffeic, ferulic, coumaric, vanillic and syringic acids.

**Figure 1:**
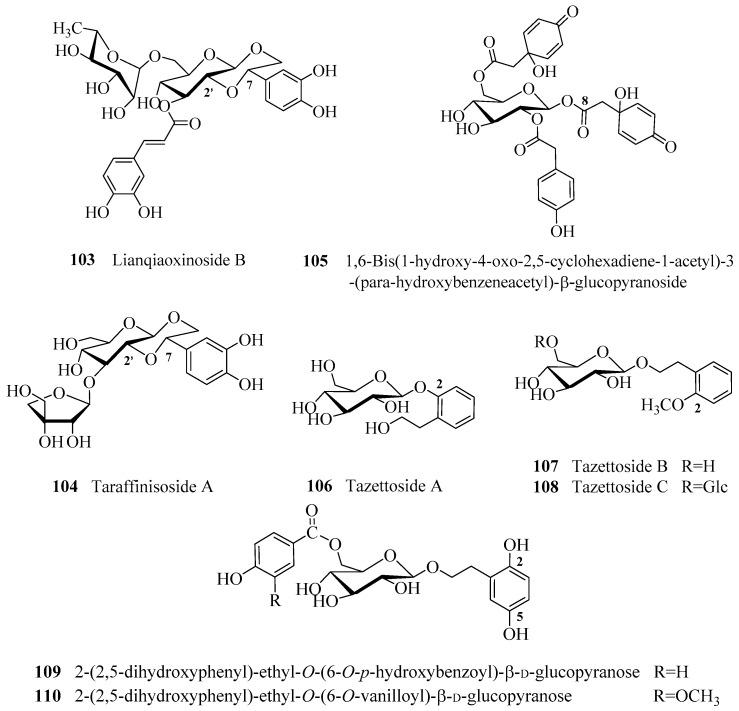

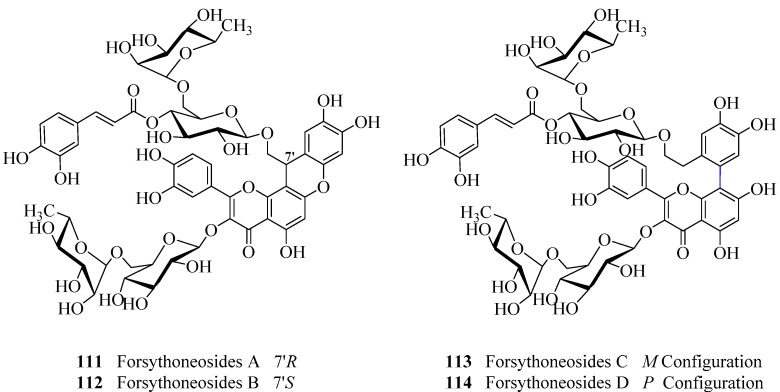

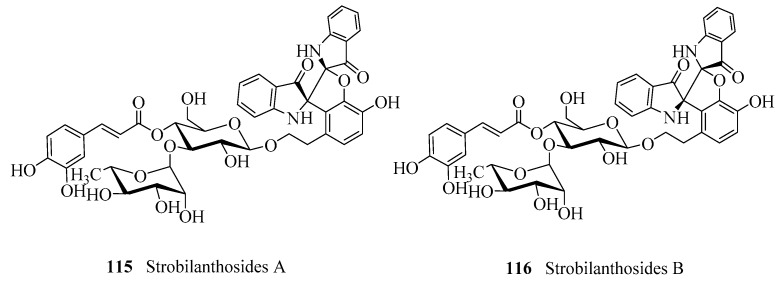
The new phenylethanoid glycosides with varied core structures or special substituents.

Figure 1 illustrates the new PhGs having varied core structures or special substituents. PhGs with a 7,2′-epoxy moiety are rare in the plant kingdom, e.g., compound **103** from *Forsythia suspensa* which is reported to possess antioxidant as well as antimicrobial activities [49], and compound **104** from *Tarphochlamys affinis* which was shown to have antioxidant as well as anti-HBV activities [50]. Compound **105** from *Jacaranda mimosifolia* with antioxidant activity is an example of a PhG with a substituent at C-8 [51]. Compound **106** with melanogenesis inhibitory activity as well as compounds **107** and **108** from *Narcissus tazetta* var. *chinensis* are examples of PhGs with substituents at C-2 [36]. Compounds **109** and **110** from *F. suspensa* are examples of PhGs with substituents at C-2 and C-5 of the phenylethyl moiety [20]. There were also adducts of other kinds of compound units fused to PhGs. Compounds **111**–**114** from *F. suspensa* with neuroprotective effects are four unusual adducts of a flavonoid unit fused to a phenylethanoid glycoside through a pyran ring or carbon-carbon bond [20]. Compounds **115** and **116** from *Strobilanthes cusia* are adducts of an indole alkaloid group fused to a phenylethanoid glycoside [52].

## 3. Pharmacological Activity

The pharmacological activities of the PhGs are discussed by two ways. The pharmacological activities of the new PhGs, mainly focused on the hepatoprotective, antioxidant activity, cytotoxity, anti-inflammatory and α-glucosidase inhibitory, were listed in Table 1, while the pharmacological activities of the old PhGs which are listed in Table 2 were introduced in the following.

### 3.1. Neuroprotective Effects

Parkinson’s disease is characterized by a selective degeneration of dopaminergic neurons in the substantia nigra pars compacta and consequently a reduction in striatal dopamine levels [53]. 1-Methyl-4-phenyl-1,2,3,6-tetrahydropyridine (MPTP) is known to cause Parkinsonism in rodents and non-human primates [54,55]. It can cause a partial lesion of the substantia nigra and a significant reduction in striatal dopamine levels [56], and the toxicity of MPTP depends on its biotransformation to its active metabolite 1-methyl-4-phenylpyridinium (MPP^+^) [57]. The potential neuroprotective and behavioral rescue effects of echinacoside (**117**) were evaluated in a mouse model of MPTP-induced dopaminergic neuronal damage. In which, an HPLC analysis was conducted to monitor the changes in the levels of striatal dopamine and its metabolites. The results showed that the reductions in the levels of dopamine and 3,4-dihydroxyphenylacetic acid (DOPAC) were partially prevented by pre-treatment of **117** (20 mg/kg) (dopamine, 0.86 ± 0.05 ng/mg tissue, *p* < 0.01; DOPAC, 0.93 ± 0.06 ng/mg tissue, *p* < 0.05). Tyrosine hydroxylase is the rate-limiting enzyme in dopamine biosynthesis. Immunostaining of the substantia nigra using an anti-tyrosine hydroxylase antibody demonstrated pre-treatment of **117** (20 mg/kg) for 15 days significantly reduced MPTP-induced tyrosine hydroxylase-positive dopaminergic neuron loss (*p* < 0.05). In addition, the pre-treatment with **117** can significantly reduce caspase-3 and caspase-8 activation induced by MPP^+^ in cerebellar granule neurons, which was regarded to be a possible mechanism on neuroprotection of **117** [58].

Pedicularioside A (**118**), leucosceptoside A (**119**), isoacteoside (**120**), acteoside (**121**), and arenariside (**122**) were studied to assess their effects on MPP^+^-induced cell death in rat mesencephalic neurons [59]. Compound **118** had the greatest neuroprotective effect among the five tested compounds. The pre-treatment with **118** inhibited MPP^+^-induced loss and death of dopaminergic neurons, and the immunohistochemistry results indicated that **118** inhibited expression of caspase-3 gene and cleavage of poly (ADP-ribose) polymerase in cultures exposed to MPP^+^. All suggested that the inhibition of caspase-3 gene expression of **118** protected mesencephalic neurons from MPP^+^-induced cell death.

Considerable evidence supported that oxidative stress worked as a common pathogenetic mechanism in Alzheimer’s disease (AD) [60,61]. In AD, oxidative stress was suspected to be mainly generated by β-amyloid peptide (Aβ) [62], and heme oxygenase-1 (HO-1) was a crucial factor in the response to oxidative injury, protecting neurons against Aβ-induced injury. Wang studied the neuroprotective mechanisms of **121** against Aβ_25-35_-induced cell death in PC12 cells. It showed that **121** was an activator of NF-E2-related factor 2 (Nrf2) and inducer of HO-1 expression. Compound **121** attenuated Aβ_25-35_-induced neurotoxicity by induction of HO-1 via extracellular regulated kinase (ERK) and PI3K/Akt signaling [63]. Similarly, the neuroprotective effects of salidroside (**123**) following traumatic brain injury were mediated, at least in part, through activation of the PI3K/Akt signaling pathway [64]. In another study, the neuroprotective effect of **121** on Aβ_25-35_-induced neurotoxicity in SH-SY5Y cells was investigated. A 3-[4,5-dimethylthiazol-2-yl]-2,5-diphenyl- tetrazolium bromide (MTT) reduction assay showed that 20 and 30 μg/mL of **121** significantly blocked cytotoxic effects of Aβ_25-35_ on cell viability and the result was also confirmed by calcein-AM staining assay through analysis of morphological nuclear changes and DNA fragmentation. Meanwhile, pretreatment with 20 μg/mL of **121** decreased the number of apoptotic cells and scavenged reactive oxygen species (ROS). The result indicated that **121** could protect SH-SY5Y cells against Aβ_25-35_-induced cell injury by attenuating ROS production and modulating apoptotic signal pathway through Bcl-2 family, cytochrome c, and caspase-3 [65]. Peng et al. [66] investigated the effects of **121** in improving learning and memory using a mouse model of senescence induced by a combination of D-galactose and AlCl_3_. Compound **121** was administered intragastrically at doses of 30, 60 and 120 mg/kg/day for 30 days after AD was induced. The results showed that the latency of step down was shortened in AD model mice and the number of errors decreased after treatment with all doses of **121**. Neurons and Nissl bodies in the hippocampus were increased significantly with higher doses (60 and 120 mg/kg/day) of **121**. The content of nitric oxide (NO), the activity of nitric oxide synthase and the expression of caspase-3 protein were decreased by 120 mg/kg/day of **121** compared with that in the AD model group [66]. In a previous study, anti-amnesic activities of **121** using scopolamine-induced amnesic mice with both passive avoidance and Morris water maze test were examined. In both tests, the prolonged oral treatment of **121** (0.1 and 1.0 mg/kg body weight respectively for 10 days) significantly improved the memory deficits, while, the acute treatment of **121** (1.0 and 2.5 mg/kg body weight for 1 day) showed positive effect only in the passive avoidance test [67].

As reported, the anti-apoptotic action of **117** was partially dependent on its anti-oxidative effects [68,69]. Kuang’s experiment indicated that **117** increased cell viability and decreased the apoptotic ratio by reducing ROS generation in H_2_O_2_-injured rat PC12 cell. In addition, compound **117** prevented H_2_O_2_-induced increase of the Bax/Bcl-2 ratio by down-regulating Bax protein expression and up-regulating Bcl-2 protein expression. The result suggested that **117** showed significant neuroprotective effects on H_2_O_2_-injured PC12 cell through the mitochondrial apoptotic pathway [70]. Similarly, the antioxidant property and neuroprotective effects of isocampneoside II (**131**) were studied on H_2_O_2_-induced oxidative injury in PC12 cells. Compound **131** inhibited cell apoptosis by decreasing the level of superoxide anion radical, inhibiting Bax/Bcl-2 ratio, and attenuating the decrease of superoxide dismutase (SOD) and catalase activity [71].

### 3.2. Antioxidant Activity

ROS are inevitably generated during the normal metabolism of living organisms, but excessive production leads to oxidative stress damage to cellular structures [72]. Oxidative stress is associated with the etiology of a wide range of chronic and acute disease such as malignant tumors, inflammation, cataracts, Parkinson’s and Alzheimer’s disease, hypertension, diabetes, atherosclerosis, cardiovascular diseases, cell death, and some immune disorders and the aging process [72,73]. PhGs have been reported to possess antioxidant properties. Forsythoside B (**125**), leucosceptoside B (**126**) and **121** were isolated from *Verbascum xanthophoeniceum* and exhibited potent antioxidant activities in 2, 2′-diphenyl-1-picrylhydrazyl (DPPH), oxygen radical absorbance capacity (ORAC_FL_), hydroxyl radical averting capacity (HORAC_FL_), ferric-reducing antioxidant power (FRAP) and superoxide anion radical scavenging assays [1]. In another study, Harput et al. reported calceorioside A (**127**) as well as **121** showed strong radical scavenging effects against DPPH, NO and superoxide anion radical comparable to that of known antioxidants [74]. Recently, DPPH· scavenging, anti-LP assays, ABTS^+^· scavenging, OH scavenging, superoxide anion radical scavenging, Cu^2+^-chelating and FRAP assays were used to evaluate the antioxidant activities of poliumoside (**128**), alyssonoside (**129**), brandioside (**130**), **121**, **125** and their derivatives, and the tested compounds were all screened out as antioxidants [75]. The structure-activity relationship between PhGs and their antioxidant activities indicated that the ortho-dihydroxyphenyl group was the important group, and the steric hindrance, the number as well as the position of phenolic hydroxyl were also thought to play an important effect [76].

### 3.3. Anti-Inflammatory Effect

*Pseudomonas aeruginosa* is the major pathogen implicated in sepsis and pneumonia [77,78]. Total phenylethanoid glycosides (TPG) from *Monochasma savatieri* prolonged survival rate of mice with *P. aeruginosa* or *Staphylococcus aureus* infection-induced sepsis in vivo. Meanwhile, TPG could reduce the bacterial colony-forming units in lung tissue in mice model. In addition, TPG (60–180 mg/kg) had significantly reduced xylene-induced ear edema and cotton pellet-induced granulomat formation at a dose-dependent manner. Furthermore, the treatment of TPG (1.5 g/kg) for 15 days did not cause any death of rat and no organic toxicity at the dose equal to approximately 284 times of clinical dose used [79]. It was reported that compound induced HO-1 in macrophages through p38 mitogen-activated protein kinase (MAPK)/Nrf2 signaling and decreased the release of high mobility group box 1 (HMGB1) in lipopolysaccharide (LPS)-stimulated Raw264.7 cells and in cecal ligation and puncture (CLP)-induced septic mice. In vitro, compound **121** not only inhibited the release of HMGB1, the production of inducible nitric oxide synthase and NO, but also induced HO-1 expression in a concentration-dependent manner; in vivo, it increased survival and decreased the HMGB1 levels of serum and lung in CLP-induced sepsis [80]. In another study, the anti-inflammatory activity, the anti-nociceptive activity, and the wound healing activity of **121** were studied using a carrageenan-induced hind paw edema model in vivo, a *p*-benzoquinone-induced abdominal constriction test, and incision and excision models in vivo, respectively [81]. It was previously reported that **121** was more active than ibuprofen in the writhing test (67.6% and 50.0% at equimolar doses) and showed similar effects in the tail flick (topic and oral) at equivalent dose to ibuprofen [82]. Moreover, compound **121** was found to be active in a carrageenan-induced hind paw edema model and in *p*-benzoquinone-induced writhing in mice [83]. Penido et al., revealed that **121** exhibited a potent inhibitory effect on LPS-induced total leucocyte, neutrophil and eosinophil accumulation in the pelural cavity along with a potent antiulcerogenic activity against diclofenac-induced gastric ulcers at 100 mg/kg [84]. Meanwhile, the histological scores indicated that treatment with **121** ameliorated intestinal inflammation in both acute and chronic dextran sulphate sodium-induced colitis in vivo through inhibition of oxidative burst activity [85]. Cell adhesion molecules (CAMs) play a role in the pathogenesis of atherosclerosis and inflammation. Compounds and 6-*O*-acetyl-acteoside (**132**) inhibited IL-1β-activated expression of intercellular CAM-1 and vascular CAM-1 (VCAM-1) in human umbilical vein endothelial cells (HUVECs). Compounds **121** and dose-dependently inhibited VCAM-1 gene promoter activity in IL-1β-activated HUVECs and their inhibition on IL-1β-activated expression of CAMs was manifested by decreased phosphorylation of ERK and c-Jun N-terminal kinase (JNK) [86]. Georgiev et al., studied anti-inflammatory properties of and forsythoside (**124**) towards human keratinocytes. Compounds **121** and **124** were both equally effective inhibitors of IL-8 release at 50 mM, with more than 90% reduction of IL-8 at spontaneous levels. Meanwhile, they significantly and dose-dependently impaired the release of IFN-γ-induced MCP-1 and IP-10 as well as significantly reduced background and IFN-γ-induced levels of IL-8 mRNA [87]. In addition, the protective effect of **123** on ethanol-induced acute gastric ulcer and H_2_O_2_-induced gastric epithelial cell damage were investigated. Intragastrical treatment with **123** inhibited the overproduction of pro-inflammatory cytokines (interleukin-6, interleukin-1β and tumor necrosis factor-α), enhanced antioxidant activity and alleviated acute gastric ulcer as well as gastric epithelial cell damage through the MAPK/NF-κB pathway [88].

### 3.4. Antibacterial and Antivirus Activity

The antimicrobial activity of TPG from *M. savatieri* was studied in vivo and in vitro. In vitro, TPG showed significant bacteriostatic properties against *S. aureus*, *P. aeruginosa*, *Escherichia coli*, *Enterococcus faecalis*, and *Streptococcus pneumoniae* at a concentration between 0.0625 and 16 mg/mL [79]. The anti-influenza virus effect of TPG from *Ligustrum purpurascens* was reported in vivo and in vitro. In vivo, C57BL/6J mice were given oral administration of TPG once daily for five successive days. TPG significantly decreased the mouse lung index (*p* < 0.05), alleviated influenza-induced lethality and clinical symptoms, and subsequently enhanced mouse survival (*p* < 0.05). In vitro, TPG inhibited influenza A virus H_1_N_1_ infection of MDCK cells in a hemagglutination assay [89]. Besides, many pure PhGs also possessed potent antibacterial activity. Compounds **121** and **125** showed considerable antibacterial activities against all strains of *S. aureus* with the minimum inhibitory concentration (MIC) values ranging from 64 μg/L to 256 μg/L. Particularly, the activities of **121** (MIC = 2.1 × 10^−4^ and 4.1 × 10^−4^ M) and **125** (MIC = 3.4 × 10^−4^ M) against SA 1199B (NorA) and XU 212 (TetK/MecA), respectively, were comparable to those of the positive control, norfloxacin (MIC = 1.0 × 10^−4^ and 2.5 × 10^−5^ M) [90]. In addition, 4′′′-*O*-acetylacteoside (**133**) and **121** possessed significant inhibition of the formation of bacterial biofilms by *E. coli* UTI89 [91]. The antifungal/antimicrobial effect of PhGs may be largely due to the presence of phenolic hydroxyls which have high affinity with proteins [92].

### 3.5. Anti-Tumor Activity

The effects of **123** on the growth of human breast cancer in vitro and in vivo were evaluated, and it was found that **123** inhibited the proliferation of breast adenocarcinoma (MCF-7) cells with half maximal inhibitory concentration (IC_50_) value of 19.48 µM, and promoted the apoptosis of MCF-7 cells in a dose-dependent manner by increasing the activity of caspase, up-regulating the Bax expression, and down-regulating the Bcl-2 expression. In addition, compound **123** significantly diminished not only the weight but also the volume of tumor (*p* < 0.05) in a nude mouse mode. Compound **123** inhibited the intracellular ROS formation and MAPK pathway activation, which may contribute to the inhibition of tumor growth [93]. Compound **121** was reported to be a potent anti-cancer drug in the treatment of fibrosarcoma metastasis. It inhibited phorbol-12-myristate-13-acetate-induced matrix metalloproteinase-9 expression via Ca^2+^-dependent calmodulin-dependent protein kinase (CaMK)/ERK and JNK/nuclear factor-𝜅B (NF-𝜅B)-signaling pathways [94]. Cytotoxic activities of **121** and **127** against human larynx epidermoid carcinoma, human rhabdomyosarcoma and human MCF-7 cell lines were determined with the IC_50_ from 36.24 µg/mL to 64.6 µg/mL, and apoptotic cell death was observed in histological analysis [74]. In another study, compounds **119**, **120**, **121**, **125**, **129**, and decaffeoyl-acteoside (**134**) from *Marrubium thessalum* were assayed by MTT and ^3^H-thymidine incorporation assays, and **120** and **121** showed tumor toxicity, while, they also showed low toxicity against peripheral blood mononuclear cells [95].

### 3.6. Immunomodulatory Effect

Autoimmune hepatitis (AIH) is a severe form of hepatitis. Studies have indicated that inflammatory cytokines and T lymphocytes play important roles in the pathogenesis of AIH [96,97]. Concanavalin A-induced hepatitis in a mouse model was regarded as the immune-mediated liver injury that resembles AIH occurring in human [98]. Hu et al., reported the intravenous (*i.v.*) injection of **123** dramatically reduced the levels of alanine aminotransferase and aspartic transaminase in the above mentioned mouse model, and partly suppressed the secretion of proinflammatory cytokines through downregulating the activity of NF-κB. Meanwhile, compound **123** altered the distribution of CD4^+^ and CD8^+^ T lymphocyte in the liver and spleen through regulating CXCL-10 and decreased the severity of liver injuries [99]. Song extracted TPG from *L. purpurascens* and tested the immune enhancement effect of the TPG using serum hemolysin antibody, phagocytosis, splenocyte antibody production, and NK cells activity assays. Mice treated with TPG showed an increase in the haemagglutination titre, the antibody production of spleen cells, MΦ phagocytosis of chicken RBCs and NK cell activity [100]. Huang et al., established a screening model of immunological activity by using dendritic cells as target cells to investigate the effects of **120** and **121** on the phenotypic and functional maturation of dendritic cells. Expressions of major histocompatibility complex (MHC) class II and costimulatory molecules were used as indicators of successful maturation, and dendritic cells treated with **120** and **121** expressed high level of class II MHC and costimulatory molecule CD86 (B7-2). In addition, increased naïve T cell stimulatory activity and decreased endocytosis further confirmed the functional maturation of dendritic cells [101].

### 3.7. Enzyme Inhibitory Activity

Prescott et al. found that **121**, teucrioside (**135**) and lamiuside A (**136**) (caffeoyl phenylethanoid glycosides) were direct calcineurin inhibitors when assayed both in the presence and absence of calmodulin using *p*-nitrophenyl phosphate as substrate [102]. In Georgiev’s study, compound **125** and the phenylethanoid fractions from the Devil’s claw cultures showed higher butyrylcholinesterase inhibitory activity than that of galanthamine [103]. Compound **131** was found to significantly inhibit recombinant human aldose reductase with an IC_50_ value of 9.72 µM. Furthermore, it inhibited sorbitol formation in a rat lens incubated with a high concentration of glucose [104]. Meanwhile, the effect of pure PhG on improving glucose tolerance was also performed in vivo and in vitro. Compounds **117** and **121** inhibited the increase in postprandial blood glucose levels in starchloaded mice at doses of 250–500 mg/kg *p.o.* and also significantly improved glucose tolerance in starchloaded mice after 2 weeks of continuous administration at doses of 125 and/or 250 mg/kg/day *p.o.* without producing significant changes in body weight or food intake. In vitro, nine of pure PhGs demonstrated potent rat lens aldose reductase inhibitory activity. In particular, 2′-acetyl-acteoside (**137**) (0.071 µM) was similar to that of epalrestat (0.072 µM), a clinical aldose reductase inhibitor [105]. In an alloxan-induced diabetic mice model, compound **123** significantly reduced fasting blood glucose, total cholesterol, triglyceride and methane dicarboxylic aldehyde levels, and at same time increased serum insulin levels, SOD, glutathione peroxidase and catalase activities [106].

### 3.8. Other Pharmacological Effects

The effect of **121** on a 42-mer amyloid β protein aggregation was examined by using the thioflavin-T assay, transmission electron microscopy, and circular dichroism spectroscopy. Compound **121** strongly inhibited the aggregation of 42-mer amyloid β protein in a dose-dependent manner [107]. In another study, compound **121** appeared an inhibitory effect on DHT-induced secretion of both free and total prostate-specific antigen at all tested concentration in an in vitro model of human prostate epithelium [108]. He et al. studied the vasorelaxant activity of **117** and the results highlighted that **117** could evoke a significant endothelium-dependent vasorelaxation action mediated through the NO-cGMP pathway in an isolated rat thoracic aorta ring [109].

## 4. Pharmacokinetics

### 4.1. Pharmacokinetics of Echinacoside (***117***) and Acteoside (***121***)

Compounds **117** and **121** are the major PhGs in Herba Cistanchis, and **117** is widely present in plants. **117** contained additional glucose linking to C-6 of core saccharide compared with **121**, and both of them exhibited good bioactivities [58,59,64,65]. In Caco-2 cell monolayer model, compounds **117**, **120** and **121** were primarily transported via poorly absorbed passive diffusion down a concentration gradient without efflux [110], which was consistent with the result that the caffeic acid conjugates permeated poorly through the Caco-2 monolayers [111]. Though the absorption of **117** was poor, it was significantly increased when **117** was combined with verapamil and clove oil both in situ and in vitro [112].

PhGs were characterized by low intestinal absorption due to their physicochemical characteristics such as molecular sizes, degrees of polymerization and solubilities [113], but it is a growing recognition that not only the absorbed PhGs but also their metabolites may contribute to their pharmacological activities [114,115]. For example, the hydrolyzing metabolites of **117** and **121,** such as hydroxytyrosol (HT) and 3-hydroxyphenylpropionic acid (3-HPP), possessed antioxidant [116,117], neuroprotective [118,119,120], and anti-inflammatory activities [121,122]. Identification of **117**’s metabolites produced by human intestinal bacteria, biliary metabolites as well as urinary and fecal ones was reported. Eight phase II metabolites of parent compound (methyl ethers, glucuronides, and minor sulfates) were isolated and identified unambiguously from rat bile sample after *i.v.* administration of **117** [123]. Unlike the metabolites in rat bile, besides the phase II metabolites of parent compound, the degradation products and their glucuronic acid, sulfate, and methyl conjugations were identified in rat urine and feces [124]. PhGs were reported to be transformed by the intestinal bacteria before being absorbed into blood [125]. Compound **117** was found to be stable in simulated gastric juice and intestinal juice, whereas it could be metabolized by intestinal bacteria. Thirteen metabolites of compound **117** and five possible metabolic pathways, including hydroxylation, dehydroxylation, reduction, deglycosylation, and acetylation were identified using UPLC-quadrupole time-of-flight mass spectrometry (UPLC-Q-TOF-MS) with MS^E^ technology and MetaboLynx software. In addition, HT and 3-HPP were found to be bioactive metabolites of **117**. The fact that HT and 3-HPP possessed biological functions similar to those of **117**, could potentially explain that **117** has prominent bioactivity but poor bioavailability [126].

Up to thirty-five metabolites were observed in the urine samples of rats orally administered with compound **121**, through processes of oxidization, glucuronidation, sulfation, and methylation. Interestingly, the metabolism of **121** occurred much quickly than those of the degradation products, while the concentrations of metabolites from the degradation products were much higher than that of **121** [127]. The metabolic profiles of **121** produced by human or rat intestinal bacteria or intestinal enzyme in vitro were also reported. 3-HPP (56.13%), HT (24.77%) and reduction **121** or its isomers (18.07%) were the main products of **121** produced by the action of human bacteria, while 3-HPP (55.75%) and **134** (36.31%) were the main products of **121** produced by rat bacteria. The content of metabolite produced by intestinal enzyme was lower than that produced by intestinal bacteria, which indicated that intestinal bacteria had more impact on the absorption and metabolism of **121** than that of intestinal enzyme [128,129].

Further pharmacokinetic study was also reported to offer suitable references in PhGs’ clinical applications. Compound **121** was absorbed fast with low peak area, and the integral area under drug concentration-time curve (AUC) was small, which indicated few **121** were absorbed into the circulatory system. Its moderate elimination made less possibility of organ injury [130,131]. Interestingly, double peaks were seen from concentration-time curve of **121** in rat plasma [131,132]. And its absolute bioavailability was 0.12% [133]. The absorption of **117** was also fast with lower peak area, and elimination was faster than that of **121**, but the absolute bioavailability of **117** with a value of 0.83% was a bit higher than that of **121** [134]. The different results of **117** and **121** may be ascribed to their structural difference, i.e., more than one glucose existed in the C-6 of **117**, which meant that **117** was easier to be hydrolyzed and resulted in lower peak area as well as faster elimination. Another issue was that the value of *T*_max_ of **117** obtained from the study performed by Yang [135] was prolonged to 90 min compared to Jia’s study [134]. Jia’s study was conducted in three groups of rats collected to develop a full pharmacokinetic profile whereas in Yang’s study the full pharmacokinetic profile was obtained from a group of rats.

With the development of analysis and extraction technology, more and more sensitive and specific methods were reposted. What’s more, simultaneous determination of more than one chemical marker and their pharmacokinetic studies were also reported. The microemulsion liquid chromatography (MELC) method [136] and the two-phase hollow fiber liquid phase microextraction coupled with a magnetofluid technique [137] for simultaneous determination of **117**, **120**, **121** and tubuloside B (**139**) in rat plasma after oral administration of *Cistanche salsa* extract by HPLC were developed. In the MELC method, the calibration curve for the four PhGs was linear in the range of 10–1000 ng/mL with the correlation coefficients greater than 0.9994. The RSDs of intra-day and inter-day precision were below 8.64% and the limits of detection (LOD) for the four PhGs were 0.4–1.3 ng/mL (S/N = 3). Under the MELC method, the calibration curve for PhGs was linear in the range of 0.1–100 ng/mL with correlation coefficients greater than 0.9996. The RSDs of intra-day and inter-day precision were below 8.74% and the LOD for the four PhGs were 8–15 pg/mL (S/N = 3).

### 4.2. Pharmacokinetics of Salidroside (***123***) and p-Tyrosol

Guo et al. [138] established an HPLC-tandem mass spectrometry method to determine **123** and its aglycone metabolite *p*-tyrosol in rat plasma after *i.v.* (50 mg/kg) and intragastric gavage (*i.g.*) (100 mg/kg) administration of **123** to rats. Both **123** and *p*-tyrosol were detected after *i.v.* administration, the *T*_1/2_ of elimination phase was prolonged 1.34 fold to 1.64 ± 0.30 h for *p*-tyrosol, comparing with that of 0.70 ± 0.21 h for **123**. According to AUC_0-__∞_ data, about 2% of **123** was present as the aglycone metabolite, *p*-tyrosol, in plasma. On the other hand, only **123** was detected after *i.g.* administration, with *T*_1/2_ value at 1.32 ± 0.22 h. It indicated that **123** was eliminated quickly after both *i.v.* and *i.g.* administrations in vivo. In addition, **123** may metabolize to *p*-tyrosol after *i.g.* administration, whereas it may be further metabolized to other metabolites, and resulted in undetectable *p*-tyrosol in the plasma sample [138]. The speculation was verified by Hu’s experiment, in which **123** and its deglycosylation phase I metabolite *p*-tyrosol were further metabolized to glucuronidation and sulfation products and mainly excreted through the urine excretion pathway [139]. Later, Guo’s research team studied the metabolism of **123** and *p*-tyrosol in liver tissues after *i.v.* administration of **123** (50 mg/kg) to rats, in which *T*_1/2_ values were 0.54 ± 0.06 h and 0.92 ± 0.03 h for **123** and *p*-tyrosol, respectively. In addition, the higher mean residence time and clearance (CL) values of *p*-tyrosol suggested that *p*-tyrosol was eliminated more slowly than **123** in liver tissues [140]. These differences in the pharmacokinetics parameters of **123** and *p*-tyrosol might be attributed to their chemical properties. Compound **123** is made up of aglycone *p*-tyrosol and a glucopyranose through glycosidic linkage, which makes it more water-soluble and consequently leads to a more rapid elimination than its aglycone [141]. The same goes for the deconjugation of flavonoid glucuronides, which could also lead to prolonged circulation and enhanced bioactivity in in vitro studies [141,142]. The elimination of **123** in rats was fast but slow (*T*_1/2_, 120.0 min) in beagle dogs after a single *i.v.* at a dose of 75 mg/kg [143], which indicated species difference existed in metabolism of **123**. In addition, different dosages and administrative patterns might affect the bioavailability of **123**. The bioavailability of **123** was calcaluted as 51.97% at dosages of 100 mg/kg *i.g.* and 50 mg/kg *i.v.* administration [138], 32.1% at dosages of 12 mg/kg oral and *i.v.* administration [144] and 98.0% at dosages of 25 mg/kg oral and 5 mg/kg *i.v.* administration [145].

### 4.3. Pharmacokinetics of Forsythoside (***124***)

It was found that **124** was rapidly absorbed into the circulation system and reached its peak concentration (*C*_max_, 122.2 ± 45.4 ng/mL) at around 20 min following oral administration (100 mg/kg) in rats. Similarly, its absolute bioavailability was also quite low with a value of 0.5% [146]. The potential hydrolysis in the gastrointestinal tract, poor permeability through the intestinal epithelial membrane and first-pass effect in the liver might be responsible for the low bioavailability of **124**. Though the low permeability of **124** leads to low oral bioavailability of **124** [147,148], water-soluble chitosan at dosage of 50 mg/kg improved the bioavailability of **124** and the antioxidant activity in vivo [149]. Meanwhile, the metabolism and bioactivity studies of **124** also showed that its metabolites HT and dihydrocaffeic acid exhibited more potent anti-complement, antimicrobial and antiendotoxin effects than itself [150].

The pharmacokinetic characteristics of **124** in dogs after *i.v.* administration of 5, 10 or 20 mg/kg of **124**, respectively, were also reported. The AUC and *C*_max_ increased proportionally with the increasing doses, but CL and *T*_1/2_ were not dose-dependence. The result that **124** was eliminated quickly and its *T*_1/2_ was short, clued to that **124** should be given by continuous *i.v.* infusion to maintain clinical effect. Meanwhile, the relative large values of distribution volume (V_d_, 1.10–1.90 L/kg) suggested that **124** was easily to distribute into tissues, which was beneficial to the treatment of infectious diseases in tissues [151]. It’s worth noting that *T*_1/2_ and V_d_ of **124** in dogs were different from those in rats [152], the species difference existed and deep reason needed further investigation.

The pharmacokinetics and hepatobiliary excretion of **124** in rats were also reported. The results indicated that hepatobiliary excretion was an important excretion path for **124**. Furthermore, the disposition of **124** in blood and bile suggested that there was rapid exchange and equilibration between the blood and hepatobiliary systems [153].

A comparative pharmacokinetic study of **124** in rats after administration of Shuang-huang-lian (SHL) solutions via *i.v.*, peroral or intratracheal routes was reported [154]. The plasma concentration of **124** reached the peak at 45 min with *C*_max_ of 35.0 ± 7.1 ng/mL after oral administration of 1000 mg/kg SHL solutions. The absolute bioavailability was determined to be 0.72% for **124**. Whereas, the intratracheal delivery produced the peak plasma concentration within 5 min, and the absolute bioavailability of **124** via pulmonary route was determined to be 25.8%. The absorption characteristic of **124** from the respiratory tract was distinct from that via the peroral route. Compared to peroral administration, pulmonary delivered chemical markers more rapidly and thoroughly absorbed.

### 4.4. Pharmacokinetics of Other PhGs

Plantamajoside (***138***) was a unique compound that characterizes *Plantago asiatica*. The mean plasma concentration-time profile of **138** in rats after oral administration of 10 g/kg (dry herb weight equivalent) was reported. The pharmacokinetic results showed **138** was quickly absorbed in rats with the time of 16.7 min to maximum plasma concentration (*C*_max_, 172.3 ± 35.1 ng/mL). The elimination rate constants was 0.28 ± 0.01 L/h and *T*_1/2_ was 2.46 ± 1.0 h [132]. Pharmacokinetics of tyrosol galactoside (**140**) following oral and *i.v.* administration both at a dose of 60 mg/kg were performed [155]. The oral bioavailability of **140** was about 27.9%, which was similar to that of compound **123** calculated at dosages of 12 mg/kg oral and *i.v.* administration [144].

## 5. Conclusions

The structural diversity of PhGs and the resulting biological properties, including neuroprotective, anti-inflammatory, antioxidant, anti-aging, memory enhancement, antibacterial, antivirus, cytotoxic, immunomodulatory, and enzyme inhibitory effects are attractive to those engaged in drug discovery. Pure PhGs and herbs rich in PhGs have been shown to possess multiple medical functions in vitro and in vivo. The poor permeability through the intestinal epithelial membrane, hydrolysis by enzymes in the gastrointestinal tract, and interaction with the enriched intestinal bacteria are the three possible reasons for the poor bioavailability of PhGs. Metabolic studies revealed that PhGs could be presumed to act as prodrugs, which were easily hydrolyzed in vivo and mainly metabolized into degradation products. There is a growing recognition that not only the absorbed parent PhGs, but also their metabolites may have the potential to be the effective ingredients, while most pharmacokinetic studies have focused on prototype compounds rather than their metabolites, so intensive studies of metabolite pharmacokinetics are required to shed light on the mechanisms underlying their systemic health effects of these compounds and confirm their clinical potential.

## Figures and Tables

**Table 1:**
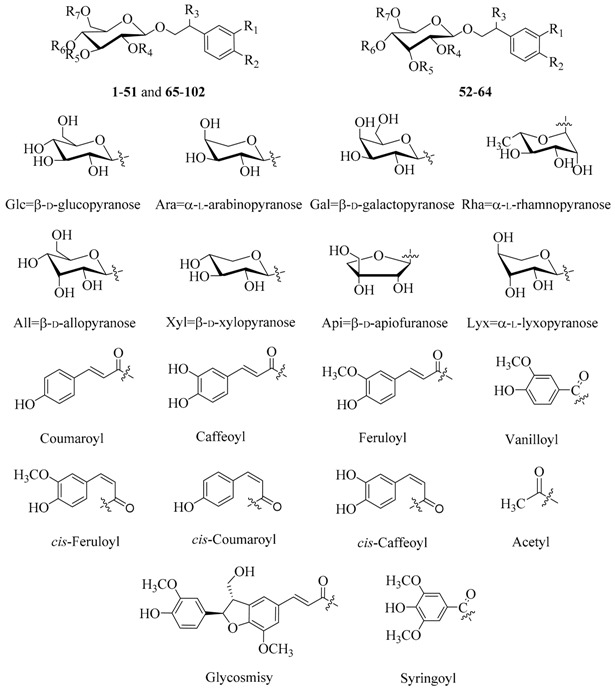
The new phenylethanoid glycosides with typical phenethyl alcohol moieties attached to a β-glucopyranose/β-allopyranose.

No.	Compounds	R_1_	R_2_	R_3_	R_4_	R_5_	R_6_	R_7_	Source	Bioactivity	Reference
**1**	Acanmontanoside	OH	OH	H	H	4-*O*-Syringoyl-Rha	Caffeoyl	H	*Acanthus montanus*	- ^a^	[5]
**2**	Kansanoside A	H	H	H	Gal	H	H	Xyl	*Asclepias syriaca*	- ^a^	[6]
**3**	Bacomoside A	OH	OH	=O	*p*-hydroxy-benzoyl	H	H	H	*Bacopa monniera*	- ^b^	[7]
**4**	Bacomoside B_1_/B_2_	OH	OH	OCH_3_	Caffeoyl	H	H	H	*B. monniera*	Inhibitory effects on Aβ_42_ aggregation	[7]
**5**	Himaloside A	OCH_3_	OH	H	Acetyl	Glc(1→4)Rha	Caffeoyl	H	*Boschniakia himalaica*	Antibacterial activity	[8]
**6**	Himaloside B	OH	OH	H	H	H	H	*cis*-Caffeoyl	*B. himalaica*	Antibacterial activity	[8]
**7**	*Z*-Tubuloside D	OH	OH	H	Acetyl	2,3,4-tri-*O*-Acetyl-Rha	Coumaroyl	Glc	*Cistanche violacea*	- ^a^	[9]
**8**	Cistanoside J	OCH_3_	OH	H	Acetyl	Rha	H	Feruloyl	*C. deserticola*	Anti-inflammatory activity	[10]
**9**	Cistanoside K	OCH_3_	OH	H	Acetyl	Rha	H	Caffeoyl	*C. deserticola*	Anti-inflammatory activity	[10]
**10**	Cistanoside L	OCH_3_	OCH_3_	H	H	Rha	H	Feruloyl	*C. deserticola*	- ^b^	[10]
**11**	Cistanoside M	OCH_3_	OH	H	H	Rha	H	Coumaroyl	*C. deserticola*	Anti-inflammatory activity	[10]
**12**	Cistanoside N	OCH_3_	OH	H	Acetyl	Rha	H	3-*O*-Glc-Caffeoyl	*C. deserticola*	Anti-inflammatory activity	[10]
**13**	Kankanoside J_1_/J_2_	OH	OH	OCH_3_	Acetyl	Rha	Caffeoyl	H	*C. tubulosa*	- ^a^	[11]
**14**	Kankanoside K_1_/K_2_	OH	OH	OCH_3_	H	Rha	Caffeoyl	Glc	*C. tubulosa*	Hepatoprotective activity	[11]
**15**	Kankanoside H_1_/H_2_	OH	OH	H	Acetyl	Rha	*trans*/*cis*-Coumaroyl	Glc	*C. tubulosa*	- ^a^	[12]
**16**	Kankanoside I	H	H	H	H	Rha	Caffeoyl	Glc	*C. tubulosa*	- ^a^	[12]
**17**	Cistansinenside B	OH	OCH_3_	H	Acetyl	Rha	Caffeoyl	Rha	*C. sinensis*	- ^a^	[13]
**18**	Bunginoside A	H	OH	H	5-*O*-glycosmisyl-Api	H	H	H	*Clerodendrum bungei*	- ^a^	[14]
**19**	3″,4″-di-*O*-acetylmartynoside	OH	OCH_3_	H	H	3,4-di-*O*-Acetyl-Rha	Feruloyl	H	*C. bungei*	- ^b^	[14]
**20**	β-d-Glucopyranoside,1″-*O*-(7*S*)-7-(3-methoxyl-4-hydroxy-phenyl)-7-methoxyethyl-3″-α-l-rhamn-opyranosyl-4″-[(8*E*)-7-(3-metho-xyl-4-hydroxy-phenyl)-8-propenoate]	OCH_3_	OH	OCH_3_	H	Rha	Feruloyl	H	*Cirsium setosum*	- ^b^	[15]
**21**	β-d-Glucopyranoside,1″-*O*-(7*S*)-7-(3-methoxyl-4-hydroxy-phenyl)-7-methoxyethyl-3″-α-l-rhamn-opyranosyl-4″-[(8*E*)-7-(4-hydrox-yphenyl)-8-propenoate]	OCH_3_	OH	OCH_3_	H	Rha	Coumaroyl	H	*C. setosum*	Hepatoprotective effect	[15]
**22**	Peiioside B	OH	OH	H	H	Rha	H	Api	*Callicarpa peii*	-^a^	[16]
**23**	Purpureaside D	OH	OH	H	H	H	Feruloyl	Rha	*Digitalis purpurea*	Antioxidant activity	[17]
**24**	Purpureaside E	OH	OH	H	H	Glc	Feruloyl	Rha	*D. purpurea*	Antioxidant activity	[17]
**25**	Forsythenside K	OH	OH	H	H	H	Coumaroyl	Rha	*Forsythia suspensa*	Antiviral activity	[18]
**26**	Lianqiaoxinside A	OH	OH	H	H	Caffeoyl	H	Rha	*F. suspensa*	Antibacterial activity	[19]
**27**	2-(3,4-Dihydroxyphenyl)-2-oxo-ethyl-*O*-α-l-hamnopyranosyl-(1→6)-(4-*O*-caffeoyl)-β-d-glucopyranoside	OH	OH	=O	H	H	Caffeoyl	Rha	*F. suspensa*	- ^b^	[20]
**28**	Forsythoside A 4′-*O*-β-d-glucopyranoside	OH	OH	H	H	H	4-*O*-Glc-Caffeoyl	Rha	*F. suspensa*	- ^b^	[20]
**29**	Isoforsythoside	OH	OH	H	H	Caffeoyl	H	Rha	*F. suspensa*	Antioxidant and antibacterial effects	[21]
**30**	Forsythoside H	OH	OH	H	Caffeoyl	H	H	Rha	*F. suspensa*	- ^a^	[22]
**31**	Forsythoside I	OH	OH	H	H	Caffeoyl	H	Rha	*F. suspensa*	- ^a^	[22]
**32**	Forsythoside J	OH	OH	H	Caffeoyl	H	H	Xyl	*F. suspensa*	- ^a^	[22]
**33**	Calceolarioside *A*-2′-α-l-rhamnopyranoside	OH	OH	H	Rha	H	Caffeoyl	H	*Fraxinus mandschurica*	- ^a^	[23]
**34**	3′′′-*O*-Methylcampneoside I	OH	OH	OCH_3_	H	Rha	Feruloyl	H	*Incarvillea compacta*	Hepatoprotective and antioxidant effects	[24]
**35**	6′-*O*-(*cis*-1,4-Dihydroxycyclohex-nacetyl) acteoside	OH	OH	H	H	Rha	Caffeoyl	*cis*-1,4-Dihydroxy-cyclohexanacetyl	*Jacaranda caucana*	Antioxidant capacity	[25]
**36**	6′-*O*-(1-Hydroxy-4-oxo-cyclohexanacetyl) acteoside	OH	OH	H	H	Rha	Caffeoyl	1-Hydroxy-4-oxo-cyclohexanacetyl	*J. caucana*	Antioxidant capacity	[25]
**37**	Fucatoside A	OH	OH	H	Api	H	Caffeoyl	H	*Lantana fucata*	- ^b^	[26]
**38**	Fucatoside B	OH	OH	H	Xyl	Api	Caffeoyl	H	*L. fucata*	- ^b^	[26]
**39**	Fucatoside C	OH	OH	H	Api	Api	Caffeoyl	H	*L. fucata*	Anti-inflammatory effect	[26]
**40**	Raduloside	OH	OH	H	H	Api	Caffeoyl	Api(1→4)Xyl	*L. radula*	- ^b^	[27]
**41**	Leonoside E	OCH_3_	OH	H	H	Ara(1→2)Rha	H	H	*Leonurus japonicus*	Hepatoprotective activity	[28]
**42**	Leonoside F	OCH_3_	OH	H	H	Rha	H	Glc	*L. japonicus*	Hepatoprotective activity	[28]
**43**	β-(4-Hydroxyphenyl) ethyl-4-*O*-*E*-caffeoyl-*O*-[β-d-apiofuranosyl-(1→2)]-β-d-glucopyranoside	H	OH	H	Api	H	Caffeoyl	H	*Lepisorus contortus*	Cytotoxity	[29]
**44**	β-(3,4-Dihydroxyphenyl) ethyl-6-*O*-*E*-caffeoyl-*O*-[β-d-apiofuranosyl-(1→2)]-β-d-glucopyranoside	OH	OH	H	Api	H	H	Caffeoyl	*L. contortus*	Cytotoxity	[29]
**45**	β-(3,4-Dihydroxyphenyl) ethyl-4-*O*-*E*-caffeoyl-*O*-[β-d-apiofuranosyl-(1→2)]-β-d-glucopyranoside	OH	OH	H	Api	H	Caffeoyl	H	*L. contortus*	- ^b^	[29]
**46**	β-(3,4-Dihydroxyphenyl) ethyl-3-*O*-*E*-caffeoyl-*O*-[β-d-apiofuranosyl-(1→2)]-β-d-glucopyranoside	OH	OH	H	Api	Caffeoyl	H	H	*L. contortus*	Cytotoxity	[29]
**47**	β-(4-Hydroxyphenyl) ethyl-3-*O*-*E*-caffeoyl-*O*-[β-d-apiofuranosyl-(1→2)]-β-d-glucopyranoside	H	OH	H	Api	Caffeoyl	H	H	*L. contortus*	- ^b^	[29]
**48**	Lagotiside A	OH	OH	H	H	4-*O*-CH_3_-Xyl	Caffeoyl	H	*Lagotis brevituba*	- ^a^	[30]
**49**	Yulanoside A	OH	OH	H	Rha	Rha	Caffeoyl	Glc(1→4)Glc	*Magnolia salicifolia*	- ^a^	[31]
**50**	Yulanoside B	OH	OH	H	H	Rha	Caffeoyl	Glc(1→4)Glc	*M. salicifolia*	- ^a^	[31]
**51**	2′-Rhamnoechinacoside	OH	OH	H	Rha	Rha	Caffeoyl	Glc	*M. salicifolia*	*α*-Glucosidase inhibitory effect and cytotoxicity	[31,32]
**52**	Magnoloside D	OH	OH	H	Rha	H	H	Caffeoyl	*M. officinalis*	Antioxidant activity, α-glucosidase inhibitory effect and cytotoxicity	[32,33]
**53**	Magnoloside E	OH	OH	H	Api	H	H	Caffeoyl	*M. officinalis*	Antioxidant activity, α-glucosidase inhibitory effect and cytotoxicity	[32,33]
**54**	Magnoloside F	OH	OH	H	Rha	H	Caffeoyl	Glc	*M. officinalis*	α-Glucosidase inhibitory effect and cytotoxicity	[32]
**55**	Magnoloside G	OH	OH	H	Api	H	Caffeoyl	Glc	*M. officinalis*	Cytotoxicity	[32]
**56**	Magnoloside H	OH	OH	H	Api	Caffeoyl	H	Glc	*M. officinalis*	α-Glucosidase inhibitory effect and cytotoxicity	[32]
**57**	Magnoloside I	OH	OH	H	Api	Coumaroyl	H	Glc	*M. officinalis*	*α*-Glucosidase inhibitory effect	[32]
**58**	Magnoloside J	OH	OCH_3_	H	Rha	Caffeoyl	H	Glc	*M. officinalis*	Cytotoxicity	[32]
**59**	Magnoloside K	OH	OH	H	Rha	Feruloyl	H	Glc	*M. officinalis*	α-Glucosidase inhibitory effect and cytotoxicity	[32]
**60**	Magnoloside L	OH	OH	H	Api	Caffeoyl	H	H	*M. officinalis*	Cytotoxicity	[32]
**61**	Magnoloside M	OH	OH	H	Rha	H	Caffeoyl	H	*M. officinalis*	- ^a^	[32]
**62**	Magnoloside N	OH	*O*-Glc	H	Rha	Caffeoyl	H	Glc	*M. officinalis*	- ^a^	[32]
**63**	Magnoloside O	OH	OH	H	H	H	H	Glc(1→4)Rha(1→4)-Syringoyl	*M. officinalis*	Cytotoxicity	[32]
**64**	Magnoloside P	OH	OH	H	H	H	H	Glc(1→4)Rha(1→4)-Vanilloyl	*M. officinalis*	Cytotoxicity	[32]
**65**	Savaside A	OH	OH	OH	Rha	H	H	Caffeoyl	*Monochasma savatieri*	Anticomplement activity	[34]
**66**	Savaside B	OH	OH	OH	Rha	H	Caffeoyl	H	*M. savatieri*	Anticomplement activity	[34]
**67**	Savaside C	OH	OH	OH	Rha	H	Feruloyl	H	*M. savatieri*	Anticomplement activity	[34]
**68**	Savaside D	OH	OH	OH	Rha	H	H	Coumaroyl	*M. savatieri*	Anticomplement activity	[34]
**69**	Savaside E	OH	OH	OH	Rha	H	H	Feruloyl	*M. savatieri*	Anticomplement activity	[34]
**70**	Rashomoside A	OH	OH	H	H	Xyl	Caffeoyl	Glc	*Meehania urticifolia*	- ^b^	[35]
**71**	Tazettoside D	H	OCH_3_	H	H	H	H	Glc	*Narcissus tazetta* var. *chinensis*	Melanogenesis inhibitory activity	[36]
**72**	3-Hydroxy-4-methoxy-β-phenylethoxy-*O*-[2,3-di-acetyl-*α*-L-rhamnopyranosyl-(1→3)]-4-*O*-*cis*-feruloyl-[β-d-apiofuranosyl-(1→6)]-β-d-glucopyranoside	OH	OCH_3_	H	H	2,3-di-*O*-Acetyl-Rha	*cis*-Feruloyl	Api	*Phlomis umbrosa*	- ^a^	[37]
**73**	3′′′-Acetyl-*O*-betonyoside D	OH	OCH_3_	H	H	3-*O*-Acetyl-Rha	Feruloyl	Api	*P. umbrosa*	Cytotoxic activity	[38]
**74**	2′′′, 3′′′-Diacetyl-*O*-betonyoside D	OH	OCH_3_	H	H	2,3-di-*O*-Acetyl-Rha	Feruloyl	Api	*P. umbrosa*	Cytotoxic activity	[38]
**75**	3′′′,4′′′-Diacetyl-*O*-betonyoside D	OH	OCH_3_	H	H	3,4-di-*O*-Acetyl-Rha	Feruloyl	Api	*P. umbrosa*	Cytotoxic activity	[38]
**76**	Stewartiiside	OH	OH	H	H	Api(1→4)Rha	Caffeoyl	Rha	*P. stewartii*	*α*-Glucosidase inhibitory activity	[39]
**77**	2-(3-Hydroxy-4-methoxyphenyl) ethanol 1-*O*-[α-l-rhamnopyranosyl-(1→2)-β-d-glucopyranoside]	OH	OCH_3_	H	Rha	H	H	H	*Plantago depressa*	- ^a^	[40]
**78**	2-(3,4-Dihydroxyphenyl) ethyl 3-*O*-β-d-allopyranosyl-6-*O*-caffeoyl-β-d-glucopyranoside	OH	OH	H	H	All	H	Caffeoyl	*P. asiatica*	Antioxidative effect	[41]
**79**	Isocassifolioside	OH	OH	H	Rha	Rha	H	Caffeoyl	*Ruellia tuberosa*	Antioxidant activity	[42]
**80**	Lavandulifolioside B	OCH_3_	OH	H	H	Ara(1→2)Rha	4-*O*-CH_3_-Feruloyl	H	*Stachys lavandulifolia*	- ^b^	[43]
**81**	Poliumoside B	OH	OH	H	H	Ara(1→2)Rha	Caffeoyl	Rha	*Teucrium polium*	Antioxidant activity	[44]
**82**	1-(3,4-Dihydroxyphenylethyl)-*O*-α-l-lyxopyranosyl-(1→2)-*α*-l-hamnopyranosyl-(1→3)-6-*O*-transferuloyl-β-d-glucopyranoside	OH	OH	H	H	Lyx(1→2)Rha	H	Feruloyl	*T. chamaedris*	Antioxidant activity	[45]
**83**	Chionoside A	OH	OH	H	Ara	Glc	Feruloyl	H	*Veronica thomsonii*	- ^a^	[46]
**84**	Chionoside B	OH	OCH_3_	H	Ara	Glc	Feruloyl	H	*V. thomsonii*	- ^a^	[46]
**85**	Chionoside C	OH	OH	H	Ara	6-*O*-Feruloyl-Glc	Caffeoyl	H	*V. thomsonii*	- ^a^	[46]
**86**	Chionoside D	OH	OH	H	Ara	Glc	Caffeoyl	Glc	*V. thomsonii*	- ^a^	[46]
**87**	Chionoside E	OH	OH	H	Ara	Glc	Feruloyl	Glc	*V. thomsonii*	- ^a^	[46]
**88**	Chionoside F	OH	OH	H	Ara	Glc	Caffeoyl	Rha	*V. thomsonii*	- ^a^	[46]
**89**	Chionoside G	OH	OCH_3_	H	Glc	Glc	Caffeoyl	H	*V. pulvinaris*	- ^a^	[46]
**90**	Chionoside I	OH	OCH_3_	H	Glc	Glc	Feruloyl	H	*V. thomsonii* and *V. pulvinaris*	- ^a^	[46]
**91**	Isochionoside J	OH	OH	H	H	Glc(1→2)Glc	H	Caffeoyl	*V. thomsonii*	- ^a^	[46]
**92**	Isoaragoside	OH	OH	H	Ara	Glc	H	Caffeoyl	*V. thomsonii*	- ^a^	[46]
**93**	Isochionoside K	OH	OCH_3_	H	Ara	Glc	H	Caffeoyl	*V. thomsonii*	- ^a^	[46]
**94**	Isochionoside A	OH	OH	H	Ara	Glc	H	Feruloyl	*V. thomsonii*	- ^a^	[46]
**95**	Isochionoside G	OH	OCH_3_	H	Glc	Glc	H	Caffeoyl	*V. pulvinaris*	- ^a^	[46]
**96**	Isochionoside I	OH	OCH_3_	H	Glc	Glc	H	Feruloyl	*V. thomsonii* and *V. pulvinaris*	- ^a^	[46]
**97**	Helioside A	OH	OH	H	Ara	Glc	Caffeoyl	Xyl	*V. lavaudiana*	- ^a^	[47]
**98**	Helioside B	OH	OH	H	Ara	6-*O*-Caffeoyl-Glc	Caffeoyl	Xyl	*V. lavaudiana*	- ^a^	[47]
**99**	Helioside C	OH	OH	H	Ara	Glc	Feruloyl	Xyl	*V. lavaudiana*	- ^a^	[47]
**100**	Helioside D	OH	OH	H	Ara	6-*O*-Coumaroyl-Glc	Caffeoyl	H	*V. raoulii*	- ^a^	[48]
**101**	Helioside E	OH	OH	H	Ara	6-*O*-Caffeoyl-Glc	Caffeoyl	H	*V. raoulii*	- ^a^	[48]
**102**	Helioside F	OH	OH	H	Xyl	Glc	Caffeoyl	Glc	*V. hulkeana*	- ^a^	[48]

^a^ Not determined; ^b^ Show no activities at the given pharmacological models.

**Table 2:**
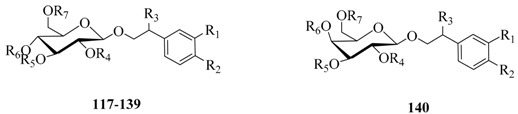
The old phenylethanoid glycosides whose pharmacological effects were reported after 2008.

No.	Compounds	R_1_	R_2_	R_3_	R_4_	R_5_	R_6_	R_7_
**117**	Echinacoside	OH	OH	H	H	Rha	Caffeoyl	Glc
**118**	Pedicularioside A	OH	OH	H	H	Api	Caffeoyl	Rha
**119**	Leucosceptoside A	OH	OH	H	H	Rha	Feruloyl	H
**120**	Isoacteoside	OH	OH	H	H	Rha	H	Caffeoyl
**121**	Acteoside (Verbascoside)	OH	OH	H	H	Rha	Caffeoyl	H
**122**	Arenariside	OH	OH	H	H	Rha	Caffeoyl	Xyl
**123**	Salidroside	H	OH	H	H	H	H	H
**124**	Forsythoside (Forsythiaside/Forsythoside A)	OH	OH	H	H	H	Caffeoyl	Rha
**125**	Forsythoside B	OH	OH	H	H	Rha	Caffeoyl	Api
**126**	Leucosceptoside B	OH	OCH_3_	H	H	Rha	Feruloyl	Api
**127**	Calceorioside A	OH	OH	H	H	H	Caffeoyl	H
**128**	Poliumoside	OH	OH	H	H	Rha	Caffeoyl	Rha
**129**	Alyssonoside	OH	OH	H	H	Rha	Feruloyl	Api
**130**	Brandioside	OH	OH	H	Acetyl	Rha	Caffeoyl	Rha
**131**	Isocampneoside II	OH	OH	OH	H	Rha	H	Caffeoyl
**132**	6-*O*-Acetylacteoside	OH	OH	H	H	Rha	Caffeoyl	Acetyl
**133**	4′′′-*O*-Acetylacteoside	OH	OH	H	H	4-*O*-Acetyl-Rha	Caffeoyl	H
**134**	Decaffeoylacteoside	OH	OH	H	H	Rha	H	H
**135**	Teucrioside	OH	OH	H	H	Lyx(1→2)Rha	Caffeoyl	H
**136**	Lamiuside A	OH	OH	H	H	Gal(1→2)Rha	Caffeoyl	H
**137**	2′-Acetylacteoside	OH	OH	H	Acetyl	Rha	Caffeoyl	H
**138**	Plantamajoside	OH	OH	H	H	Glc	Caffeoyl	H
**139**	Tubuloside B	OH	OH	H	Acetyl	Rha	H	Caffeoyl
**140**	Tyrosol galactoside	H	OH	H	H	H	H	H
